# Psoriasiform Dermatitis in a COVID-19 Patient

**DOI:** 10.1155/2022/1820673

**Published:** 2022-12-16

**Authors:** Marlyn Wu, Shereen Teymour, Robin Ashinoff, Hira Ghani

**Affiliations:** ^1^Hackensack Meridian Palisades Medical Center, Palisades, NJ, USA; ^2^Nassau University Medical Center, Long Island, NY, USA

## Abstract

Psoriasis is a chronic inflammatory papulosquamous disorder which affects around 2% of the world's population. A peak exacerbation in psoriatic symptoms was noted during COVID-19 due to lack of access to dermatological care mixed with heightened emotional stress during the pandemic. This case report describes a 52-year-old admitted male patient who sustained a diffuse rash on multiple areas of his body a week prior to testing positive for COVID-19. We explore plausible causes for the occurrence of the rash, discuss our treatment plan, include relevant clinical pictures, and review published literature to examine conditions that present similarly to the rash seen in our patient. It is crucial for dermatologists to be able to discern various systemic manifestations associated with cutaneous lesions, such as the one seen in this patient, in order to make an accurate and prompt diagnosis. A better understanding of the association between COVID-19 infection and psoriasiform lesions is needed for improving the prognostic and therapeutic outcomes in patients.

## 1. Introduction

Psoriasis is a chronic inflammatory papulosquamous disorder which affects around 2% of the world's population [1, 2]. COVID‐19 is a highly contagious respiratory infection caused by severe acute respiratory syndrome coronavirus 2 (SARS-CoV2) [3]. The World Health Organization officially declared COVID-19 a pandemic on March 2020 [3]. Psoriasis is noted to be a more common dermatological disease manifestation in patients with COVID-19. Lack of access to dermatologic care during the peak of COVID-19 pandemic and emotional stress resulting from the global disease outbreak significantly contributed to exacerbation of psoriatic symptoms in those suffering from this condition [4].

In this case report, we describe a patient who sustained a diffuse rash on his upper extremities, trunk, and groin one week prior to testing positive for COVID-19. We explore plausible causes for the occurrence of the rash, discuss our treatment plan, include relevant clinical pictures, and review published literature to examine conditions that present similarly to the rash seen in our patient.

## 2. Case

A 52-year-old admitted male with no significant past medical or surgical history was evaluated for a 2-week history of an intensely pruritic, diffuse rash starting on his upper extremities and extending to his trunk. Using over the counter topical steroids provided him with minimal symptomatic relief. There was no recent use of new medications. He was seen in the emergency department (ED) for gradual onset of fever, malaise, and shortness of breath, which prompted testing for COVID-19, human immunodeficiency virus (HIV), and syphilis. While his HIV and syphilis tests were negative, he tested positive for COVID-19. The patient was treated with IV fluids, acetaminophen, piperacillin-tazobactam, and vancomycin, and was admitted for further evaluation.

**Figure 1:**
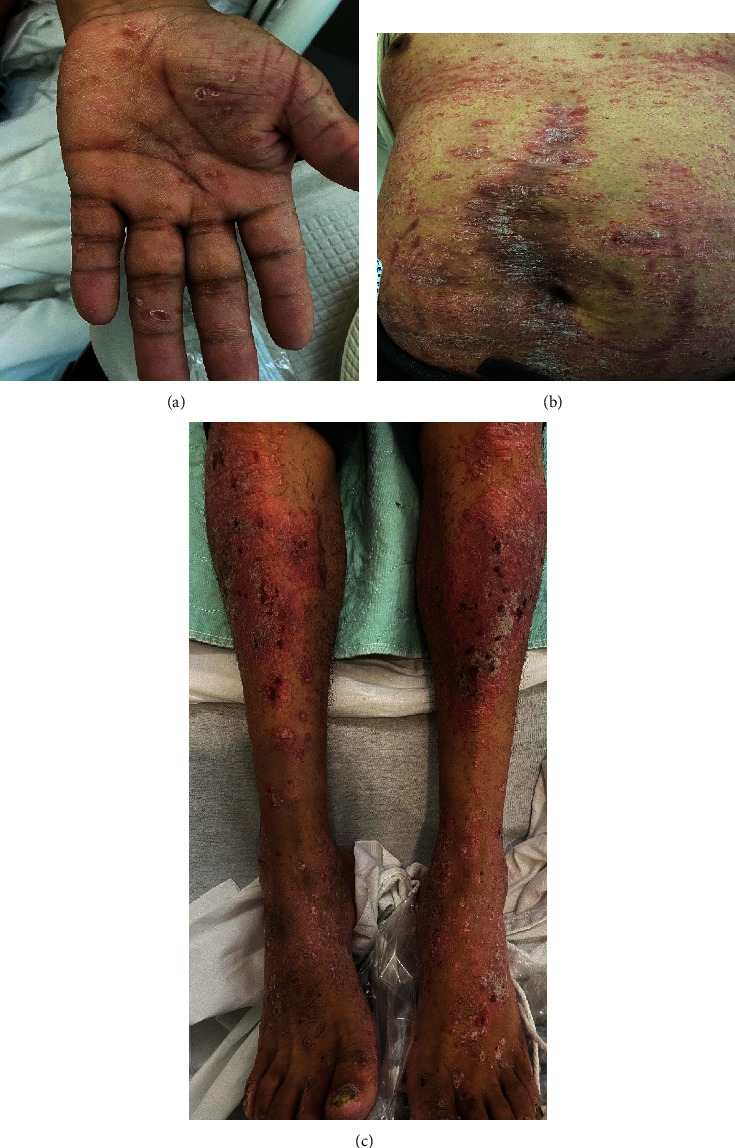
(a–c) Psoriasiform dermatitis. Clinical images depicting diffuse sharply demarcated pink plaques with white scale on the left palm, abdomen, and bilateral lower extremities.

Upon physical exam in our dermatology clinic, diffuse salmon pink papules and plaques with overlying white scales were identified on the trunk, groin, and upper and lower extremities (Figures 1(a)–1(c)). In addition, punctate scaly papules with erythema on were noted on the palms and koebnerization was observed on the patient's old hernia repair surgical site on the abdomen. A 4 mm punch biopsy of left lower abdomen revealed psoriasiform epidermal hyperplasia, hypogranulosis, and areas of confluent parakeratosis. While intraepidermal neutrophils and eosinophils were not seen, a mild perivascular mononuclear inflammatory infiltrate was appreciated. Histopathological features were consistent with psoriasiform dermatitis and psoriasis vulgaris. A negative PAS silver stain confirmed against any fungal etiology. The absence of plasma cell infiltrates decreased the possibility of secondary syphilis as a differential diagnosis.

The patient was treated with triamcinolone 0.1% ointment twice a day to affected areas for 2 weeks. Upon follow-up, a complete resolution of lesions was noticed.

## 3. Discussion

Given the clinical findings of our patient, the most likely diagnosis appears to be psoriasiform dermatitis triggered by an underlying viral (COVID-19) etiology. Various factors can trigger psoriasis in genetically predisposed individuals or exacerbate the disease when it is in remission [5]. COVID‐19 patients may exhibit features of a hyperinflammatory state, such as significantly elevated biomarkers of inflammation (CRP, ferritin), cytokines, cardiac and muscle injury, liver and kidney dysfunction, and hypercoagulation in patients with severe COVID19 [5].

It is postulated that psoriasis is a T lymphocyte-mediated disease in which activation of pathogenic T cells results in cutaneous inflammation by inducing hyperproliferation of keratinocytes. Dermal dendritic cells release certain cytokines, including interleukin-12 (IL-12) and interleukin-23 (IL-23), which results in T helper cell stimulation of epidermal hyperkeratosis and decreased apoptosis [6]. While a multitude of factors can cause psoriasis, there has been an increasing evidence suggesting the role of certain viruses such as hepatitis C, HIV, human papillomavirus (HPV), SARS-CoV2, and Zika in inducing or exacerbating psoriasis [7]. Kutlu and Metin reported a case of psoriasis exacerbation in a COVID-19 patient upon treatment with hydroxychloroquine and oseltamivir [7]. Although these drugs may worsen psoriasis, the onset of severe psoriasis within a short time frame may indicate that COVID-19 infection itself plays an crucial role in the pathogenesis of psoriasis. The presence of inflammatory cytokines, including IL-2, IL-7, and IL-10; granulocyte colony-stimulating factor; interferon-induced protein 10; monocyte chemokine 1; macrophage inflammatory protein 1; and tumor necrosis factor, have been reported to be potentially associated with the exacerbation of psoriasis in patients infected with SARS-CoV2 [7]. While we can positively identify the presence of some common cytokines mediating the underlying pathophysiology of both viral infections and psoriasis, more research is needed to better understand the pathogenesis of psoriasis induced by COVID-19 infection.

## 4. Conclusion

While many factors contribute to PSD, an underlying viral etiology such as COVID-19 or HIV infections may have a significant role in causing psoriasiform lesions in immunocompromised individuals. It is likely that the rash observed in our patient was secondary to COVID-19 infection, since no other underlying etiology could positively be identified. It is crucial for dermatologists to be able to discern various systemic manifestations associated with cutaneous lesions, such as the one seen in this patient, in order to make an accurate and prompt diagnosis. A better understanding of the association between COVID-19 infection and psoriasiform lesions is needed for improving the prognostic and therapeutic outcomes in patients.

## Data Availability

No data were used to support this study.

## References

[1] LeBoit P. E. (1992). Dermatopathologic findings in patients infected with HIV. *Dermatologic Clinics*.

[2] Wick M. R. (2017). Psoriasiform dermatitides: a brief review. *Seminars in Diagnostic Pathology*.

[3] Elmas ÖF., Demirbaş A., Kutlu Ö (2020). *Dermatologic Therapy*.

[4] Kutlu O., Metin A. (2020). Dermatological diseases presented before COVID-19: are patients with psoriasis and superficial fungal infections more vulnerable to the COVID-19?. *Dermatologic Therapy*.

[5] Henry B. M., de Oliveira M. H. S., Benoit S., Plebani M., Lippi G. (2020). Hematologic, biochemical and immune biomarker abnormalities associated with severe illness and mortality in coronavirus disease 2019 (COVID-19): a meta-analysis. *Clinical Chemistry and Laboratory Medicine*.

[6] Grän F., Kerstan A., Serfling E., Goebeler M., Muhammad K. (2020). Current developments in the immunology of psoriasis. *Yale J Biol Med*.

[7] Teng Y., Xie W., Tao X. (2021). Infection-provoked psoriasis: induced or aggravated (review). *Experimental and Therapeutic Medicine*.

